# Supplementary material for: Hormone-Independent Mouse Mammary Adenocarcinomas with Different Metastatic Potential Exhibit Different Metabolic Signatures
Source: Biomolecules. 2020 Aug 27;10(9):1242. doi: 10.3390/biom10091242 (PMC7563858; doi:10.3390/biom10091242)
Supplement: Supplementary file 1 [file biomolecules-10-01242-s001.zip › biomolecules-886999-supplementary/biomolecules-886999 supp1/supplementary_materials_description_revised.pdf]

Supporting information

Figure S1. Representative ^{1}H NMR spectra (500 MHz) of MPA-induced hormone-independent tumours extracts: a) polar extract obtained from tumour A (section A2) excluding water (5.09-4.68 ppm) and TSP (0.13-0.00 ppm) regions; assignment: (1) Isoleucine, (2) Leucine, (3) Valine, (4) Lactate, (5) Threonine, (6) Alanine, (7) Lysine, (8) Acetate, (9) Proline, (10) Glutamate, (11) Methionine, (12) Acetone, (13) Pyruvate, (14) Succinate, (15) Citrate, (16) Aspartate, (17) Creatine, (18) Phosphocreatine, (19) Ethanolamine, (20) Choline, (21) Phosphocholine, (22) Glycerophosphocholine, (23) Taurine, (24) *scyllo*-inositol, (25) Glycerol, (26) Glycine, (27) Serine, (28) Uridine, (29) Adenosine/Inosine, (30) β-Glucose, (31) α-Glucose, (32) Uracil, (33) Fumarate, (34) Tyrosine, (35) Histidine, (36) Phenylalanine, (37) Hypoxanthine and (38) Formate; b) lipophilic extract obtained from tumour F (section F2), excluding chloroform (7.50-6.96 ppm), residual methanol (3.57-3.36 ppm) and TMS (0.15-0.00 ppm) regions; assignment: (1′) Total 7-lathosterol, (2′) Total cholesterol, (3′) Fatty acids, (4′) Free cholesterol, (5′) Esterified cholesterol, (6′) Oleic acid (18:1, ω-9), (7′) Linoleic acid (18:2, ω-6), (8′) Arachidonic acid (20:4, ω-6), (9′) Docosahexaenoic acid (22:6, ω-3), (10′) Phosphatidylethanolamine, (11′) Sphingomyelin, (12′) Phosphatidylcholine, (13′) Glycerophospholipids, (14′) Triglycerides, (15′) Plasmalogen. *, residual water.

Figure S2. Scores scatter plots (lipophilic extracts) for a) PCA and b) PLS-DA of ^{1}H NMR spectra from 59-2-HI and C7-2-HI tumour lines. Q^2 (cum): predictive power.

Figure S3. Relative metabolite levels (left) and average lipid characteristics (right) for tumours of a) 59-2-HI and b) C7-2-HI lines. Heatmaps show statistically significant variations ($|ES|>0.50$, ES error<75%, Wilcoxon Rank-sum p-value<0.05) Lines and columns represent tumour sections (or octants) and metabolites, respectively. Metabolite relative peak areas are represented as the ratio of normalized integrals to the highest integral value, in a color scale. Bar plots show average fatty acids unsaturation degree $[\underline{H}C{=}C\underline{H}]/[-C\underline{H}_3]$, polyunsaturation degree $[{=}CH{-}C\underline{H}_2{-}CH{=}]/[-C\underline{H}_3]$ and average chain length $([{=}CH{-}C\underline{H}_2{-}CH{=}]+[-C\underline{H}_2{-}CO]+[-CH_2{-}C\underline{H}_2{-}CH{=}]+[(C\underline{H}_2)_n])/[(\frac{2}{3}C\underline{H}_3)])$, calculated with basis on reference [44]. *: Wilcoxon Rank-sum p-value < 0.05.

Table S1. 500 MHz ^{1}H NMR assignment of metabolites present in medroxyprogesterone acetate (MPA)-induced hormone-independent breast tumours (pH 7.4 for aqueous extracts). s, singlet; d, doublet; dd, doublet of doublets; dt, doublet of triplets; t, triplet; q, quartet; m, multiplet; br, broad.
