# Supplementary material for: Hormone-Independent Mouse Mammary Adenocarcinomas with Different Metastatic Potential Exhibit Different Metabolic Signatures
Source: Biomolecules. 2020 Aug 27;10(9):1242. doi: 10.3390/biom10091242 (PMC7563858; doi:10.3390/biom10091242)
Supplement: Supplementary file 1 [file biomolecules-10-01242-s001.zip › biomolecules-886999-supplementary/biomolecules-886999 supp1/Table_S1_assignment_17Jul20_DSB.pdf]

Table S1

Metabolite	δ ¹H / ppm (multiplicity, assignment)
**Polar compounds**	
Isoleucine	0.94 (t, δ-C**H**$_3$), 1.02 (d, γ-C**H**$_3$)
Leucine	0.96 (d, δ'-C**H**$_3$), 0.97 (d, δ-C**H**$_3$), 1.72 (m, γ-C**H**)
Valine	0.99 (d, γ-C**H**$_3$), 1.05 (d, γ'-C**H**$_3$), 2.29 (m, β-C**H**)
Lactate	1.33 (d, C**H**$_3$), 4.11 (q, C**H**)
Threonine	1.33 (d, γ-C**H**$_3$), 3.58 (d, β-C**H**), 4.25 (dd, α-C**H**)
Alanine	1.48 (d, β-C**H**$_3$), 3.77 (q, α-C**H**)
Lysine	1.53 (γ-C**H**$_2$, t), 1.72 (m, δ-C**H**$_2$), 1.92 (m, β-C**H**$_2$), 3.02 (ε-C**H**$_2$, t), 3.77 (t, α-C**H**)
Acetate	1.92 (s, β-C**H**$_3$)
Proline	2.03 (m, 3-C**H**$_2$/4-C**H**$_2$), 3.35 (dt, 2-C**H**$_2$)
Glutamate	2.05 (dt, β-C**H**$_2$), 2.13 (m, β’-C**H**$_2$), 2.36 (m, γ-C**H**$_2$), 3.77 (t, α-C**H**)
Methionine	2.13 (m, β-C**H**$_2$/ε-C**H**$_3$), 2.65 (t, γ-C**H**$_2$), 3.83 (dd, α-C**H**)
Glutamine [a]	2.14 (m, β-C**H**$_2$), 2.46 (m, γ-C**H**$_2$), 3.77 (t, α-C**H**)
Acetone	2.24 (s, α-C**H**$_3$)
Pyruvate	2.39 (s, C**H**$_3$)
Succinate	2.41 (s, α-C**H**$_2$/β-C**H**$_2$)
Citrate	2.52 (d, α-C**H**/β-C**H**), 2.67 (d, α’-C**H**/β’-C**H**)
Aspartate	2.70 (dd, β-C**H**), 2.82 (dd, β'-C**H**), 3.90 (dd, α-C**H**)
Creatine	3.04 (s, N-C**H**$_3$), 3.93 (s, N-C**H**$_2$)
Phosphocreatine	3.06 (s, C**H**$_3$), 3.94 (s, C**H**$_2$)
Ethanolamine	3.15 (t, C**H**$_2$-NH_2), 3.83 (t, C**H**$_2$-OH)
Choline	3.21 (s, N(C**H**$_3$)$_3$), 3.53 (m, β-C**H**$_2$-N), 4.07 (m, α-C**H**$_2$-OH)
Phosphocholine	3.22 (s, N(C**H**$_3$)$_3$), 3.60 (m, N-C**H**$_2$), 4.17 (m, PO_3-C**H**$_2$)
Glycerophosphocholine	3.23 (s, N(C**H**$_3$)$_3$), 3.68 (m, β′-C**H**$_2$-N$(CH_3)_3$) 4.33 (m, α’-C**H**$_2$ - PO_4^-)
Taurine	3.26 (t, C**H**$_2$-SO_3), 3.42 (t, NH_2-C**H**$_2$)
myo-inositol [b]	3.28 (t, 5-C**H**), 3.53 (dd, 1-C**H**/3-C**H**), 3. 63 (t, 4-C**H**/6-C**H**), 4.07 (t, 2-C**H**)
scyllo-inositol	3.36 (s, C**H**)
Glycerol	3.55 (dd, 1-C**H**$_2$/3-C**H**$_2$), 3.63 (dd, 1-C**H**$_2$/3-C**H**$_2$)
Glycine	3.56 (s, α-C**H**$_2$)
Serine	3.84 (dd, α-C**H**), 3.95 (dd, β-C**H**$_2$), 3.99 (dd, β′-C**H**$_2$)
Uridine	4.23 (t, 3’-CH ribose), 4.36 (t, 2’-CH ribose), 5.89 (d, 1′-CH ribose), 5.90 (d, 5-CH ring), 7.86 (d, 6-CH ring)
Adenosine/Inosine	4.28 (q, 4’-CH ribose), 4.44 (dd, 3’-CH ribose), 6.10 (d, 1’-CH ribose), 8.24 (s, 2-CH ring), 8.35 (s, 8-CH ring)
β-Glucose	4.65 (d, 1-C**H**)
α-Glucose	5.24 (d, 1-C**H**)
Uracil	5.81 (d, 5-CH ring), 7.52 (d, 6-CH ring)
Fumarate	6.52 (s, **H**C=C**H**)
Tyrosine	6.91 (d, 3-CH/5-H ring), 7.20 (d, 2-CH/6-H ring)
Histidine	7.08 (s, 4-CH ring), 7.85 (s, 2-CH)
Phenylalanine	7.33 (m, 2-CH/6-CH ring), 7.39 (m, 4-CH ring), 7.43 (m, 3-CH/5-CH ring)
Hypoxanthine	8.20 (s, 2-C**H** ring), 8.22 (s, 8-C**H**)
Formate	8.46 (s, HO-**H**C=O)
**Lipophilic compounds**	
Total 7-lathosterol	0.55 (18-C**H**$_3$, s)
Total cholesterol	0.68 (18-C**H**$_3$, s), 0.86 (26-C**H**$_3$, d), 0.87 (27-C**H**$_3$, d), 0.91 (21-C**H**$_3$, d), 1.12 (multiple cholesterol protons, m), 1.48 (multiple cholesterol protons, m), 1.84 (multiple cholesterol protons, m), 1.96 (7–C**H**$_2$/8–C**H**, br)
Fatty acids	0.88 (C**H**$_3$$(CH_2)_n$, t), 1.25 ((C**H**$_2$)$_n$, m), 1.30 (=CH-C**H**$_2$-$CH_2(CH_2)$-, m), 1.64 (-C**H**$_2$-$CH_2$-CO-, m), 2.10 (–C**H**$_2$–CH=CH–, m), 2.30 (-C**H**$_2$-CO, m), 5.35 (-**H**C=C**H**-, m)
Free cholesterol	1.01 (19-C**H**$_3$, s), 3.53 (3-C**H**, m)

Esterified cholesterol	1.02 (19-C**H**$_3$, s), 4.61 (3-C**H**, m)
Oleic acid (18:1, ω-9)	2.01 (-CH$_2$-C**H**$_2$-CH=, m)
Linoleic acid (18:2, ω-6)	2.05 (-CH$_2$-C**H**$_2$-CH=, m), 2.77 (=CH-C**H**$_2$-CH=, t)
Arachidonic acid (20:4, ω-6)	2.05 (-CH$_2$-C**H**$_2$-CH=, m), 2.82 (=CH-C**H**$_2$-CH=, m)
Docosahexaenoic acid (22:6, ω-3)	2.82 (=CH-C**H**$_2$-CH=, m)
Phosphatidylethanolamine	3.14 (–CH$_2$–C**H**$_2$–NH$_2$, br)
Sphingomyelin	3.30 (-N$^+$(C**H**$_3$)$_3$, s), 3.77 (N-C**H**$_2$, br), 5.68 (-CH$_2$-C**H**=CH-CHOH–, m)
Phosphatidylcholine	3.33 (-N$^+$(C**H**$_3$)$_3$, s), 3.77 (N-C**H**$_2$, br)
Glycerophospholipids	3.95 (3-C**H**$_2$ of glycerol, m), 4.37 (PO-(1-C**H**$_2$) **of glycerol**, m), 5.21 (2-C**H** of glycerol, m)
Triglycerides	4.14 (1-C**H**$_2$/3-C**H**$_2$ of glycerol, dd), 4.29 (1'-C**H**$_2$/3'-C**H**$_2$ of glycerol, dd), 5.26 (2-C**H** of glycerol, m)
Plasmalogen	5.90 (O-C**H**=CH, d)

[a] observed more clearly in C7-2-HI tumours; [b] tentative assignment, as identification is hindered by signal overlap particularly in 59-2-HI tumours.
