# Supplementary figures and images for: Hormone-Independent Mouse Mammary Adenocarcinomas with Different Metastatic Potential Exhibit Different Metabolic Signatures
Source: Biomolecules. 2020 Aug 27;10(9):1242. doi: 10.3390/biom10091242 (PMC7563858; doi:10.3390/biom10091242)

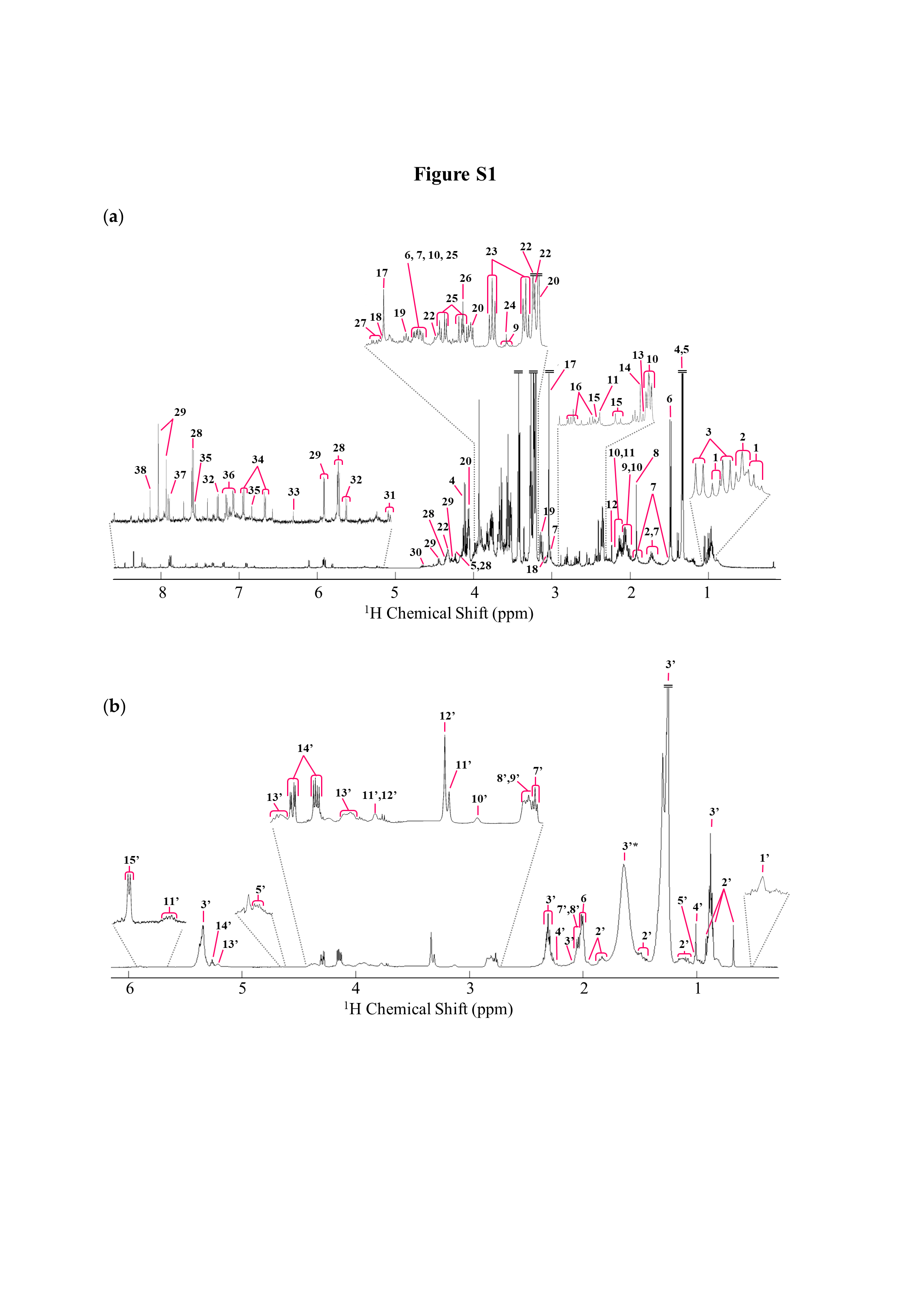

Supplement: Supplementary file 1 [file biomolecules-10-01242-s001.zip › biomolecules-886999-supplementary/biomolecules-886999 supp1/Figure_S1_spectra_AE_LE.png]

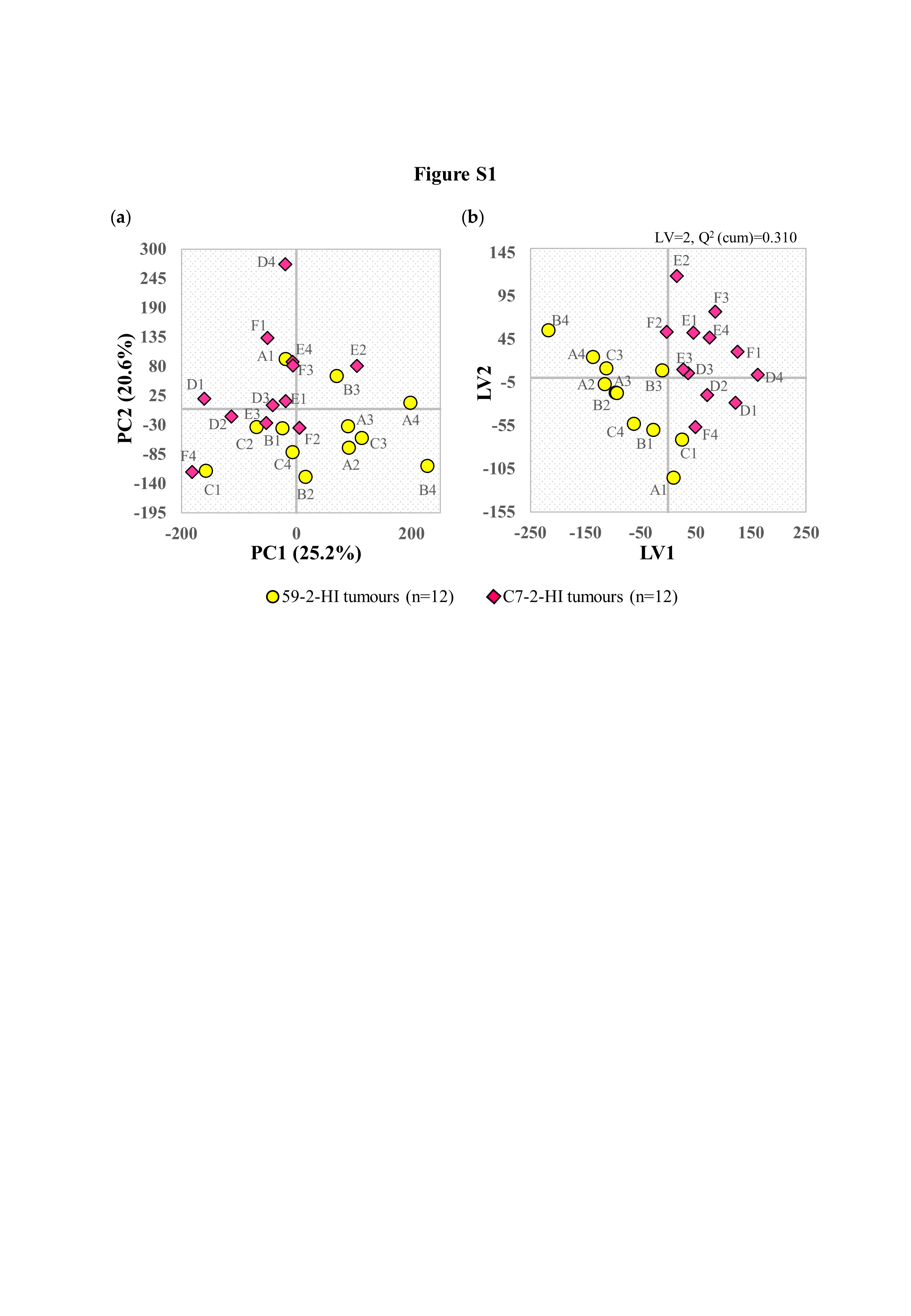

Supplement: Supplementary file 1 [file biomolecules-10-01242-s001.zip › biomolecules-886999-supplementary/biomolecules-886999 supp1/Figure_S2_LE_PCA_PLSDA.png]

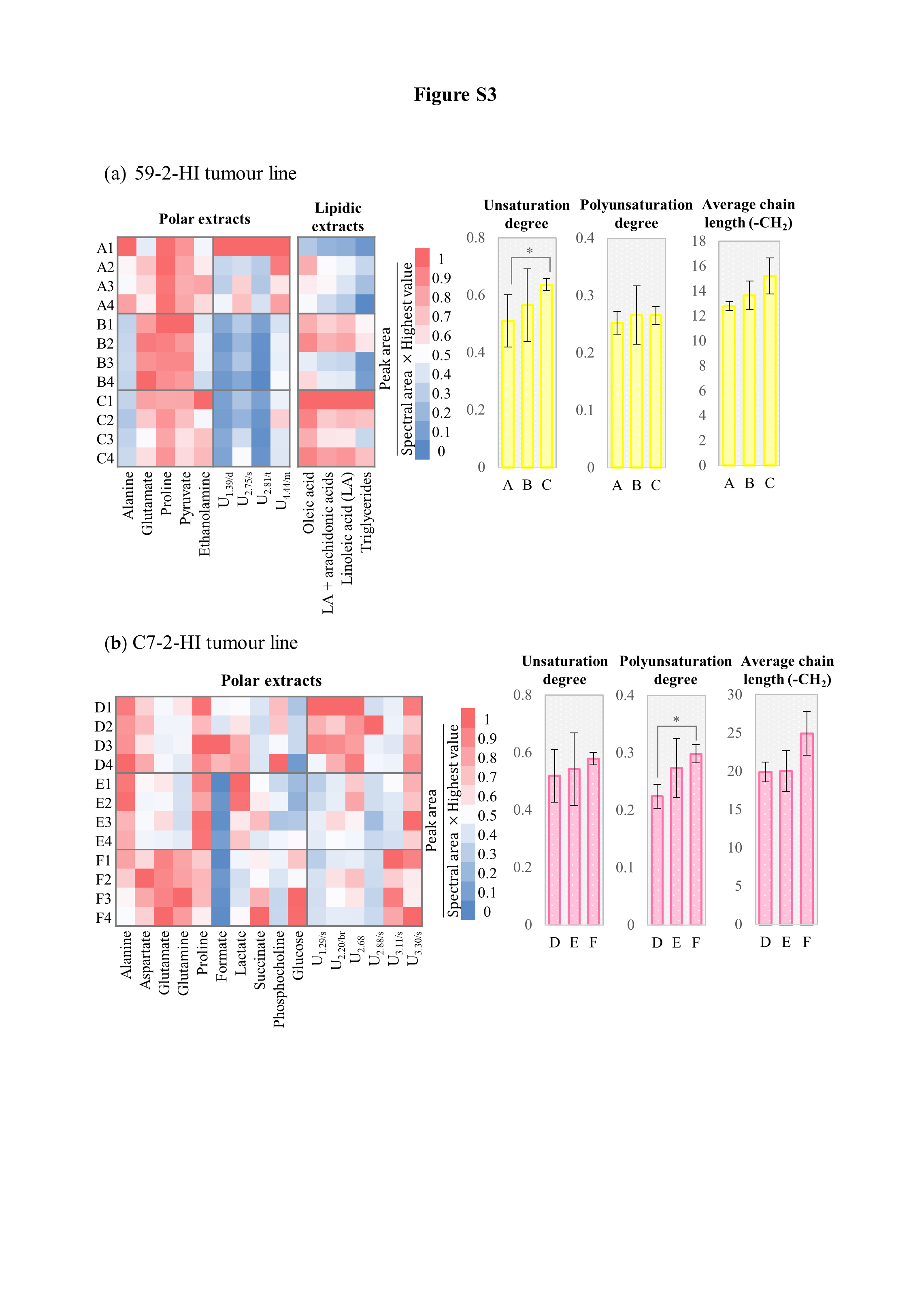

Supplement: Supplementary file 1 [file biomolecules-10-01242-s001.zip › biomolecules-886999-supplementary/biomolecules-886999 supp1/Figure_S3_REVISED.png]
